# SlMYC2 interacted with the *SlTOR* promoter and mediated JA signaling to regulate growth and fruit quality in tomato

**DOI:** 10.3389/fpls.2022.1013445

**Published:** 2022-10-27

**Authors:** Yujiao Zhang, Hongyun Xing, Haoran Wang, Lan Yu, Zhi Yang, Xiangnan Meng, Pengpeng Hu, Haiyan Fan, Yang Yu, Na Cui

**Affiliations:** ^1^ College of Bioscience and Biotechnology, Shenyang Agricultural University, Shenyang, China; ^2^ School of Pharmaceutical Sciences, Sun Yat-sen University, Guangzhou, China; ^3^ Department of Foreign Language Teaching, Shenyang Agricultural University, Shenyang, China; ^4^ Key Laboratory of Protected Horticulture of Ministry of Education, Shenyang Agricultural University, Shenyang, China

**Keywords:** *Solanum lycopersicum*, JA signaling, *SlMYC2*, TOR signaling, growth and fruit quality

## Abstract

Tomato (*Solanum lycopersicum*) is a major vegetable crop cultivated worldwide. The regulation of tomato growth and fruit quality has long been a popular research topic. MYC2 is a key regulator of the interaction between jasmonic acid (JA) signaling and other signaling pathways, and MYC2 can integrate the interaction between JA signaling and other hormone signals to regulate plant growth and development. TOR signaling is also an essential regulator of plant growth and development. However, it is unclear whether MYC2 can integrate JA signaling and TOR signaling during growth and development in tomato. Here, MeJA treatment and *SlMYC2* overexpression inhibited the growth and development of tomato seedlings and photosynthesis, but increased the sugar–acid ratio and the contents of lycopene, carotenoid, soluble sugar, total phenol and flavonoids, indicating that JA signaling inhibited the growth of tomato seedlings and altered fruit quality. When TOR signaling was inhibited by RAP, the JA content increased, and the growth and photosynthesis of tomato seedlings decreased, indicating that TOR signaling positively regulated the growth and development of tomato seedlings. Further yeast one-hybrid assays showed that SlMYC2 could bind directly to the *SlTOR* promoter. Based on GUS staining analysis, SlMYC2 regulated the transcription of *SlTOR*, indicating that SlMYC2 mediated the interaction between JA and TOR signaling by acting on the promoter of *SlTOR*. This study provides a new strategy and some theoretical basis for tomato breeding.

## Introduction

Tomato (*Solanum lycopersicum*) is a major vegetable crops, and the regulation of its growth and development, as well as yield and quality, have been popular research topics. JA is not only an important plant growth regulator but also part of an important hormone signaling pathway, playing a key role in plant growth and development (Huang et al., 2017). The exogenous application of JA inhibits various aspects of seedling growth, including primary root growth, leaf expansion, and hypocotyl elongation (Wasternack and Hause, 2013; Song et al., 2014; Kim et al., 2015). Moreover, JA treatment inhibits the expansion of true leaves and cotyledons (Zhang and Turner, 2008; Chehab et al., 2012; Aleman et al., 2016).

Important components of the JA signaling pathway include the protein COI1, complex SCF^COI1^, protein JAZ, and transcription factor MYC2 (Chini et al., 2016; Li et al., 2021a; Song et al., 2022). MYC2 is the activating component of JA signaling and belongs to the transcription factor MYC basic helix loop helix (bHLH) IIIe subfamily. The MYC IIIe family in *Arabidopsis* consists of AtMYC2, AtMYC3, AtMYC4 and AtMYC5. However, in tomato, the MYC IIIe family consists of SlMYC1 and SlMYC2 (Heim et al., 2003; Chini et al., 2016; Goossens et al., 2016), and MYC2 is the main regulator of JA signaling activation (Yan et al., 2013; Xu et al., 2018; Liu et al., 2019; Min et al., 2020).

MYC2 is widely present in animals and plants and has a variety of regulatory functions (Dombrecht et al., 2007; Altmann et al., 2020). In *Arabidopsis thaliana*, MYC2 directly binds to the promoters of *PLT1* and *PLT2*, inhibiting their expression and thereby inhibiting the growth of the main roots (Chen et al., 2011). In rice, OsMYC2 regulates the expression of *OsMADS1*, a gene that regulates the development of floral organoids and activates the development of rice spikelets (Cai et al., 2014). In apple, MdMYC2 promotes the transcription of the ethylene synthesis genes *MdACS1* and *MdACO1*, which in turn promotes ethylene synthesis in fruit and promotes fruit ripening (Li et al., 2017). In addition, MYC2 is also involved in complex metabolism. MYC2 plays an important role in regulating of artemisinin biosynthesis (Shen et al., 2016), and MYC2 regulates SGA biosynthesis in tomato and potato (Cárdenas et al., 2016; Thagun et al., 2016). The MYC2 protein contains a JID domain and an acidic AD domain at its N-terminus, and it contains a bHLH-zip domain and an ACT domain at its C-terminus (Thines et al., 2007). Elevated levels of JA-Ile promote the degradation of JAZ by the SCF^COI1^ protease complex, followed by the transcriptional expression of *MYC2*, which initiates the expression of JA-responsive genes (Zhang et al., 2015; Breeze, 2019). This finding indicates that MYC2 plays an important role in JA-mediated plant metabolism and is a high-level transcriptional regulatory element in the JA signaling pathway.

MYC2 positively regulates the inhibition of hypocotyl elongation by red or far-red light and negatively regulates the inhibition of hypocotyl elongation by blue light (Biswas et al., 2003; Riemann et al., 2008; Yan et al., 2012; Yang et al., 2012; Riemann et al., 2013), suggesting that MYC2 has different functions under different conditions. Moreover, MYC2 can integrate the interaction between JA signaling and other hormone signaling to regulate plant growth and development (Li et al., 2021b). One such example is for the ABA receptor PYL6, which interacts with the JA master regulator MYC2 to regulate the transcriptional activity of *MYC2* (Aleman et al., 2016).

JA interacts with various signaling pathways to mediate plant growth inhibition. JAs promote the degradation of JAZ to activate MYC2 and release DELLA protein (Hou et al., 2010; Yang et al., 2012). EIN3 is activated by the JA-mediated breakdown of JAZ proteins and ethylene (ET)-mediated stabilization. It binds to and represses MYC2. EIN3 also transcriptionally activates *ORA59*, and ORA59 represses *MYC2* transcription. MYC2 can enhance its own transcription in the short term but repress it in the long term. EDS1 can repress MYC2 during AvrRps4-induced ETI. In addition, SA can promote JAZs degradation during ETI *via* NPR3 and NPR4. Generally, SA is an inhibitor of *MYC2* transcription. Abscisic acid (ABA) directly activates the transcription of *MYC2* and enhances the binding of the ABA receptor PYL6 to MYC2, which regulates *MYC2* transcriptional activity, and MYC2 has different effects on the *JAZ6* and *JAZ8* promoters (Aerts et al., 2021). The above findings suggest that MYC2 mediates the interaction of JA signaling with other signaling pathways (Aerts et al., 2021).

In both tomato and *Arabidopsis*, the active hormone JA-Ile promotes COI1-dependent degradation of the JAZ repressors and thereby activates the master TF MYC2. However, studies have found that the target genes downstream of MYC2 are different in tomato and *Arabidopsis*. In tomato, MYC2 positively and directly regulates the transcription of its downstream *MTFs*, which in turn regulates the expression of late wounding-responsive genes or pathogen-responsive genes. In contrast, in *Arabidopsis*, MYC2 positively regulates wounding-responsive genes while negatively regulating pathogen-responsive genes (Du et al., 2017).

Target of rapamycin (TOR) is also an important signaling pathway in regulating plant growth and development and plays a central role in integrating metabolic energy and hormone signaling (Dobrenel et al., 2016). TOR is a highly conserved serine/threonine protein kinase in eukaryotes (Boutouja et al., 2019). Recent studies have found that the plant TOR complex can coordinate energy, growth, hormones and other signals (Zhuo et al., 2020; Fu et al., 2021), enabling it to regulate plant growth and development, as well as nutrition and energy processes, through the integration of its downstream effector proteins E2Fa and SPS (Xiong et al., 2017; De Vleesschauwer et al., 2018; Brunkard et al., 2020; Fu et al., 2020; O’Leary et al., 2020; Wang et al., 2020). Studies have shown that TOR signaling can regulate the growth and development of cotton fiber through the interaction of JAZ with JA signaling (Song et al., 2017). However, MYC2 is the core factor of signaling interactions, and it is unclear whether MYC2 can integrate JA signaling and TOR signaling pathways in response to the growth and development of tomato. Our study found that the presence of a cis-acting element of SlMYC2 on the *SlTOR* promoter, so it was speculated that SlMYC2 bound to the *SlTOR* promoter to regulate downstream response genes and thereby regulate the growth and development of tomato.

In this research, we analyzed the roles of JA signaling, SlMYC2 and TOR signaling in tomato growth and development. We demonstrated that JA signaling inhibited the growth and development of tomato plants and altered fruit quality, and TOR signaling positively regulated the growth and development of tomato seedlings. In addition, yeast one-hybrid and GUS assays indicated that SlMYC2 could directly bind to the *SlTOR* promoter and positively regulate its transcription. These results suggested that MYC2 could integrate JA signaling and TOR signaling in respond to growth and development of tomato, providing a new strategy and some theoretical basis for tomato breeding.

## Materials and methods

### Determination of seed germination and tomato growth

Tomato seeds were heated in water at 55°C for 5 min, then soaked in 70% alcohol for 1 min in an ultra-clean bench, then soaked in 5% sodium hypochlorite for 5 min, washed with ddH_2_O, and then transferred into MS medium with sterile ophthalmic forceps. The substrate containing the seeds was placed in a light incubator and incubated for 3 d at 25/18°C protected from light; and then incubated under a light intensity of 20000 Lux, photoperiod of 16/8 h, and 25/18°C while germination. The germination rate, root length and hypocotyl length were observed and recorded.

Using 0.1% DMSO as a control, the seedlings were treated with RAP (10 μM), MHY1485 (5 μM), DIECA (200 μM) and MeJA (100 μM), respectively.

### DNA construction and plant transformation

The *SlMYC2*-OE and *SlMYC2*-RNAi lines were kindly provided by Dr. Chuanyou Li’s research group (Institute of Genetics and Developmental Biology Chinese Academy of Sciences). Transgenic lines of *SlMYC2*-OE and *SlMYC2*-RNAi were constructed using Gateway (Invitrogen) technology. A fragment of the *SlMYC2* open reading frame (1–400 bp) was selected. The sequence was then cloned and inserted into pCAMBIA-1301 under the control of the *CaMV35S* promoter to generate the construct pCAMBIA-1301-*SlMYC2*-RNAi. For *SlMYC2*-OE tomato plants, the full-length coding sequence of *SlMYC2* was amplified by PCR and cloned into the pGWB5 vector to generate the Pro35S:*SlMYC2*-GFP construct. The constructs were introduced into tomato cv M82 by *Agrobacterium tumefaciens*-mediated transformation. Transformants were selected based on their resistance to hygromycin. The *A. tumefaciens*-transformed T0 generation seeds were collected, and the T1 generation was screened by hygromycin and conformed to the ratio of 3:1, indicating that there were 1/3 homozygotes, and qRT−PCR was identified as homozygous and single copy. Then, the T1 generation was planted. After identification, 10 homozygous lines were selected, and the T2 generation of seeds from a single plant was collected. After hygromycin screening, no separation occurred, showing they were all homozygotes. The T3 seeds from the T2 generation were also collected, and the T4 seeds identified as homozygotes were collected through the same identification method as above. Homozygous seeds from T3 or T4 generation were all homozygotes after identification and screening. The homozygotes of T3 or T4 transgenic seedlings were used for phenotypic and molecular characterization (Du et al., 2017).

Construction and transformation of tomato *SlTOR* gene silencing by virus-induced gene silencing (VIGS) method. A pTRV-based VIGS was performed to knock out the *SlTOR* in tomato cotyledons. A 402 bp fragment within the 3’-region of the *SlTOR* cDNA was cloned into the pTRV2 vector (TRV : *SlTOR*). The gene-specific primers were listed in **Table S1**. 10 mL of each *A. tumefaciens* strain used was incubated overnight at 28°C in YEP medium supplemented with 100 mg·L^-1^ rifampicin and 50 mg·L^-1^ kanamycin. Then, 200 µL of each overnight culture was inoculated into a 20 mL portion of YEP medium containing the above antibiotics and incubated at 28°C until the culture reached the selected optical density of OD_600_ = 0.8-1.0. Induced *A. tumefaciens* strain EHA105 carrying different pTRV2-derived vectors (pTRV2 and pTRV2-*SlTOR*) was mixed with the pTRV1 *A. tumefaciens* strain EHA105 at a ratio of 1:1. The samples were spiked with 10 mM MES, 10 mM MgCl_2_, and 200 µM acetosyringone (AS) and then the germinated tomato seeds (root length = 0.2-0.6 cm) were immersed in the bacteria and vacuumed for 4 min. The seeds were sown and cultured in a growth chamber at 22°C with a 16 h light and 8 h dark photoperiod (Yu et al., 2019).

### Determination of tomato fruit quality

Fruit hardness was measured by a TMS-Pilot Precision texture analyzer (Food Technology Corporation, Virginia, USA), and the fruit color difference was measured by a color difference meter from CHROMA METER (KONICA MINOLTA SENSING INC. Japan).

Lycopene was extracted with reference to Salvia-Trujillo and McClements (Salvia-Trujillo and McClements, 2016), total phenolic compounds were extracted with reference to Toor and Savage (Toor and Savage, 2005), and total flavonoids according to Jia et al. (Jia et al., 1999), while carotenoids were extracted by the acetone method and then all were determined spectrophotometrically. The contents of pectin, vitamin C, soluble protein, soluble sugar and acidity were also determined by spectrophotometrically.

### Quantification of JA

High performance liquid chromatography (HPLC) was used to analyze the total JA content in the leaves of tomato plants.

### Website and tools for promoter analysis

The sequences of the *SlTOR* promoter were obtained from the NCBI website (http://www.ncbi.nlm.nih.gov/). The promoter sequences of *SlTOR* were analyzed by the promoter prediction website PlantCARE.

### RNA extraction and qRT−PCR

Total RNA from plants was extracted using TRIzol reagent (Gold Hi Plasmid Mini kit, CWO581M). BioDrop was used to measure the RNA concentration, and cDNA was synthesized by the FastKing cDNA First-Strand Synthesis Kit (catalog number: KR116) from Tiangen Biochemical Technology Co., Ltd. qRT−PCR was performed using a SYBR Green PCR Master Mix kit (TIANGEN, Beijing, China) on the CFX96 Touch Real-Time PCR Detection System (Bio-Rad). Primer sequences were listed in **Table S1**. The reaction system (10 μL) contained 4.5 μL of 2× SuperReal PreMix Plus, 1 μL of cDNA, 0.5 μL of forward primer, 0.5 μL of reverse primer, and finally ddH_2_O was added to 10 μL. The reaction program included 95°C for 15 min and 40 cycles of 95°C for 10 s and 60°C for 32 s. Dissolution curve program included 65°C for 5 s and 95°C for 5 min.

### GUS staining and enzyme activity assay

The CDS of *SlMYC2* and the 2000 bp promoter region of *SlTOR* were cloned using 2×Super Pfx MasterMix from Comway Century Biotechnology Co., Ltd. The *SlMYC2* was then cloned into the pRI-101-GFP vector driven by the 35S promoter to obtain the *SlMYC2* overexpression vector, and then the empty vector pRI-101-GFP and the recombinant vector *SlMYC2*-GFP were transformed into *A. tumefaciens* EHA105, respectively. The *SlTOR* promoter was cloned into the pRI-101-GUS vector to obtain a reporter gene driving GUS expression, and this vector and the recombinant vector were transformed into *A. tumefaciens*. The bacterial broth was inoculated into YEP liquid medium and incubated at 28°C with shaking at 200 rpm until OD_600_ = 0.8-1.0. Took 20 mL of different bacterial solutions into a centrifuge tube and centrifuged at 5000 rpm for 10 min, and discarded the supernatant. Resuspended and washed the bacteria with 2 mL of immersion solution (containing 10 mM MES and 10 mM MgCl_2_·6H_2_O) and centrifuged at 5000 rpm for 10 min, and discarded the supernatant. Resuspended cells in the soaking solution (containing 10 mM MES, 10 mM MgCl_2_·6H_2_O, and 200 mM AS) to make different combinations of cells with OD_600_ ≈ 1 and incubated at 28°C in the dark for 3 h. The reporter and effector were then mixed together in a 1:1 volume ratio to transform 5-week-old *Nicotiana benthamiana*. The empty pRI-101-GUS and pRI-101-GFP vectors served as controls. Primer sequences were listed in **Table S1**. The different combinations of bacterial solutions were injected into tobacco leaves using a needleless syringe and incubated overnight in the dark, then removed and incubated normally for 1 d. The injected tobacco leaves (taken for 4 d consecutive) were immersed in 20 mL of GUS staining solution, vacuum-treated in the dark for 10 min, and stained in the dark at 37°C for 24 h. The tobacco leaves were decolorized to transparency and scanned for analysis by a scanner.

Meanwhile, 0.5 g of injected tobacco leaves was placed in a 5 mL precooled centrifuge tube and set aside in liquid nitrogen. The leaves were ground to a powder using a precooled grinder, added to 2 mL of protein extract, mixed thoroughly and refrigerated at 4°C. The mixture was incubated for 1 h to fully react and then centrifuged at 2000 × g for 20 min at 4°C, removed the supernatant to a new centrifuge tube and centrifuged at 4°C and 2000 × g for another 10 min. Then 100 μL of the total protein supernatant was collected into a 1.5 mL centrifuge tube, and 900 μL of 37°C pretreated hot GUS extraction buffer was added. The tube was placed in a water bath at 37°C for 30 min before, added 200 μL to 800 μL of 0.2 M Na_2_CO_3_. The fluorescence intensity was measured using a fluorescence spectrophotometer at EM WL of 365 nm, EX WL of 455 nm and a slit of 3 nm.

### Yeast one-hybrid assay

The pGADT7 vector was digested with *EcoR* I and *BamH* I, and pAbAi was digested by *Kpn* I and *Sal* I. The carrier fragment was then recovered. Finally, the enzymatically cleaved vector fragment and the target fragment were ligated by one-step ligation (see **Table S1** for the primer sequence list).

The motifs of *SlTOR* promoter were analyzed using PlantCARE (http://bioinformatics.psb.ugent.be/webtools/plantcare/html). Based on the analysis results, the *SlTOR* promoter with a fragment size of 1856 bp was divided into three overlapping fragments, and each fragment overlapped by about 50 bp to avoid destroying the binding sites. The *SlTOR* promoter with a fragment size of 400-900 bp was cloned using 2×Super Pfx MasterMix (CWBIO, Jiangsu, China), and then promoters of *SlTOR* were inserted into the pAbAi vector to construct the baits (pAbAi-*P_SlTOR1/2/3_*). The pAbAi-*P_SlTOR_* was introduced into Y1H Gold, encapsulated on SD/-Ura medium plates, and incubated for 2-4 d at 30°C in inverted position.

The plaque was identified by PCR. The positive plaques were recoated on a medium plate of SD/-Ura containing an AbA gradient (50-500 ng·mL^-1^). The pAbAi vector with promoter was screened for AbA resistance, i.e., the lowest concentration of AbA without phage spots was screened for subsequent yeast one-hybrid assays.

AD-*SlMYC2* was transformed into a yeast strain containing the pAbAi-*P_SlTOR_* linear vector and then encapsulated on SD/-Leu medium containing with AbA at the resistance concentration, and incubated upside down at 30°C to observe plaque growth.

### ChIP−qPCR assay

*SlMYC2*-OE tomato seedlings grown to 15 d of age were used for ChIP−qPCR analysis. ChIP−qPCR assays were performed as described by Du (2017) (Du et al., 2017). The ChIP signal was analyzed using qPCR. Each ChIP value was normalized to its respective input DNA value, and the enrichment of DNA was displayed as a percentage of the input. Primer sequences were listed in **Table S1**.

### Data processing

All experiments in this study were performed at least three repetitions. Experimental data were processed using Microsoft Excel 2010 and GraphPad Prism 6 for graphing. The significance of differences was determined by ANOVA or Student’s *t* test using IBM SPSS 20 software (*P*< 0.05). The data in the graphs were the mean ± SD (standard deviation).

## Results

### JA signaling contributed to the growth and photosynthesis of tomato seedlings

**Figure 1:**
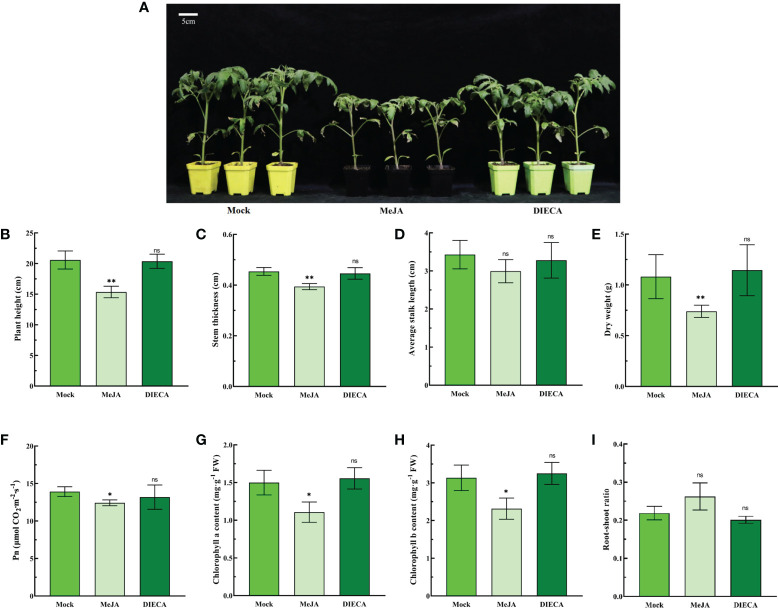
Effects of MeJA and DIECA on the growth and photosynthesis of tomato seedlings. **(A)** Phenotypic changes in 5-week-old seedlings after 1 week of treatment with 100 μM MeJA or 200 μM DIECA. **(B)** Plant height. **(C)** Stem thickness. **(D)** Average stalk length. **(E)** Dry weight. **(F)** Net photosynthetic rate (Pn). **(G)** Chlorophyll a content. **(H)** Chlorophyll b content. **(I)** Root–shoot ratio. Errors are standard deviations for three biological replicates (n=3). Differences between means were analyzed for significance using Student’s *t* test, *P< 0.05, **P < 0.01. ns, no significant difference.

JA is not only an important growth regulator but also an important signaling molecule for tomato growth and development. To investigate the regulatory effect of JA on growth and development, tomato seedlings were treated with MeJA (100 μM) and DIECA (200 μM. DIECA, JA biosynthesis inhibitor sodium diethyldithiocarbamate) (Ding et al., 2021). The results showed that MeJA inhibited plant height, stem thickness and dry weight (**Figures 1A–E**).

Photosynthesis is closely related to the dry matter accumulation of plants. The products of photosynthesis are the basis for plant growth and development, the formation of yield and quality. Therefore, the photosynthetic indexes of 5-week-old tomato seedlings were examined using a photosynthesis meter Li-6400XT. MeJA treatment significantly reduced the net photosynthetic rate (Pn) and chlorophyll a and b contents of tomato seedlings (**Figures 1F–H**). The root–shoot ratio increased slightly after MeJA treatment, but the difference was not significant compared with the control (**Figure 1I**). In addition, for all parameters, there were no significant differences between the DIECA treated and control groups (**Figure 1**).

### The transcription factor SlMYC2 contributed to the growth and photosynthesis of tomato seedlings

SlMYC2 is a key transcription factor in the JA signaling pathway. To confirm the effect of SlMYC2 on the growth and development of tomato seedlings, we first examined the expression of *SlMYC2* after MeJA and DIECA treatment. *SlMYC2* expression was significantly enhanced after MeJA treatment. The expression of *SlMYC2* decreased slightly in DIECA treatment, but it was not significant compared with the control, indicating that JA signaling could activate the expression of *SlMYC2* (**Figure S1**).

**Figure 2:**
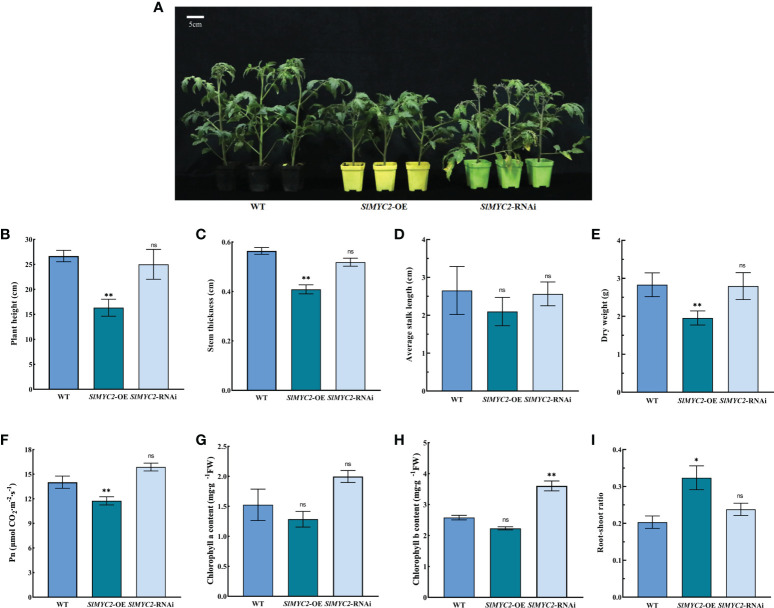
Effects of *SlMYC2* overexpression and silencing on the growth and photosynthesis of tomato seedlings. **(A)** Phenotypic of tomato seedlings (5-week-old). **(B)** Plant height. **(C)** Stem thickness. **(D)** Average stalk length. **(E)** Dry weight. **(F)** Net photosynthetic rate (Pn). **(G)** Chlorophyll a content. **(H)** Chlorophyll b content. **(I)** Root-shoot ratio. *SlMYC2*-OE, *SlMYC2* overexpression; *SlMYC2*-RNAi, *SlMYC2* silenced. Errors are standard deviations for three biological replicates (n=3). Differences between means were analyzed for significance using Student’s *t* test, **P < 0.01. ns, no significant difference.

Then, *SlMYC2* overexpression and silenced lines were used as experimental materials (**Figure S2**). The results indicated that the plant height, stem thickness and dry weight of the *SlMYC2* overexpression lines were significantly lower than those of WT, but there were no significant differences between *SlMYC2*-silenced lines and WT (**Figures 2A–E**).

In the *SlMYC2* overexpression lines, the Pn and chlorophyll a and b contents were lower than those in the WT. Among them, Pn was remarkably lower in the overexpression lines than in WT, but the chlorophyll a and b contents were not significant. The Pn and chlorophyll a and b contents in the *SlMYC2*-silenced lines showed an increasing trends; however, the chlorophyll b content was observably higher than that in the WT, while the Pn and chlorophyll a content were not significant (**Figures 2F–H**).

Nevertheless, the root–shoot ratio significantly increased in the *SlMYC2* overexpression lines, but the effect was not significant in the *SlMYC2*-silenced lines, suggesting that *SlMYC2* overexpression could enhance the number of healthy tomato seedlings (**Figure 2I**).

The above results indicated that JA and SlMYC2, key transcription factor in the JA signaling pathway, had a regulatory role in the growth and development of tomato seedlings and the net photosynthetic rate.

### Regulation of the tomato fruit quality by the transcription factor SlMYC2

Tomato fruit quality is an important indicator for its commercialization. Therefore, the relevant quality indicators of mature tomato fruits were determined after MeJA and DIECA treatment (**Figure S3**). MeJA and DIECA treatment had no significant effect on tomato fruit firmness, indicating that their effects on fruit firmness were not obvious (**Figure S3A**). Although MeJA treatment significantly reduced the tomato fruit color index and total pectin (**Figures S3B, C**), it significantly increased the contents of lycopene, carotenoid, fructose, glucose, soluble sugar, starch, soluble protein, total phenol and flavonoids (**Figures S3D–H, K, L, N, O**), while the sugar–acid ratio also increased significantly (**Figure S3J**). MeJA treatment had no significant effects on total fruit acid or vitamin C content (**Figures S3I, M**). DIECA treatment only significantly improved the content of total pectin, but significantly decreased the content of flavonoids, however, while not significantly affecting other indicators (**Figure S3**). This result suggested that increasing JA could improve the quality of tomato fruits.

**Figure 3:**
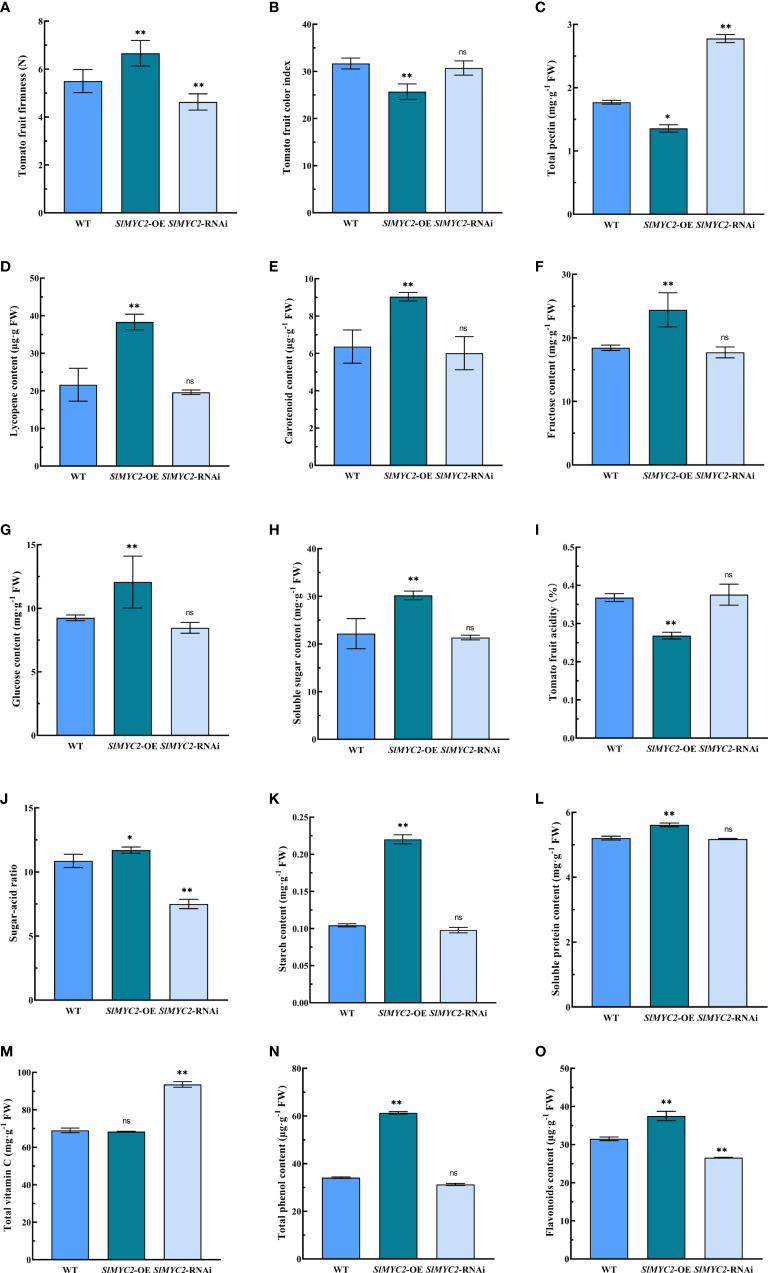
Effects of SlMYC2 overexpression and silencing on the quality of tomato fruit. **(A)** Tomato fruit firmness. **(B)** Tomato fruit color index. **(C)** Total pectin content. **(D)** Lycopene content. **(E)** Carotenoid content. **(F)** Fructose content. **(G)** Glucose content. **(H)** Soluble sugar content. **(I)** Tomato fruit acidity. **(J)** Sugar-acid ratio. **(K)** Starch content. **(L)** Soluble protein content. **(M)** Total vitamin C content. **(N)** Total phenol content. **(O)** Flavonoids conteSnltM. YC2-OE, SlMYC2 overexpression; SlMYC2-RNAi, SlMYC2 silenced. Errors are standard deviations for three biological replicates (n=3). Differences between means were analyzed for significance using Student’s t test, *P < 0.05, **P < 0.01. ns, no significant difference.

SlMYC2 is a key transcription factor in the JA signaling pathway; therefore, *SlMYC2* overexpression and *SlMYC2*-silenced lines were also used to determine tomato fruit quality (**Figure 3**). Tomato fruit firmness was significantly enhanced in the *SlMYC2* overexpression lines but significantly reduced in the silenced line (**Figure 3A**). Tomato fruit color index was significantly lower in the *SlMYC2* overexpression lines, but there was no significant difference between *SlMYC2*-silenced lines and WT (**Figure 3B**).

The color change of fruit pulp is mainly determined by the accumulation of carotenoids and lycopene. Therefore, the lycopene and carotenoids contents in tomato fruits of each group were further determined. The lycopene and carotenoid contents in mature fruits were significantly higher in the *SlMYC2* overexpression lines compared with those in the WT, but there was no significant difference in the *SlMYC2*-silenced lines (**Figures 3D, E**). The above results showed that *SlMYC2*-OE could increase the accumulation of lycopene and carotenoids and regulate fruit pulp color in mature tomato fruits.

The effects of SlMYC2 on tomato fruit were analyzed by measuring the nutrient composition of ripe fruit from the *SlMYC2* overexpression lines and *SlMYC2-*silenced lines. Total pectin content in ripe fruits from the *SlMYC2* overexpression lines was decreased significantly, whereas it increased significantly in the ripe fruits from *SlMYC2*-silenced lines (**Figure 3C**). *SlMYC2* overexpression significantly increased the contents of fructose, glucose, and soluble sugar but decreased the fruit acid content, whereas *SlMYC2* silencing had no significant effect (**Figures 3F–I**). However, *SlMYC2* overexpression increased the sugar–acid ratio in mature tomato fruits but decreased sugar–acid ratio in mature fruits of *SlMYC2*-silenced lines (**Figure 3J**). The contents of starch and total phenol in mature fruits of the *SlMYC2* overexpression lines were significantly higher than those of the control (**Figures 3K, N**), whereas the content of total vitamin C was increased with *SlMYC2* silencing (**Figure 3M**). The contents of soluble protein and flavonoids were significantly higher in mature fruits of *SlMYC2* overexpression lines, and the content of flavonoids was significantly lower in the *SlMYC2*-silenced line than in the control group, but the difference in soluble protein content was not significantly different (**Figures 3L, O**). These results revealed that JA signaling and the key transcription factor SlMYC2 were involved in the regulation of tomato fruit quality.

### Interaction effect between TOR and JA signaling on the regulation of tomato seedling growth

TOR is conserved in eukaryotes and is one of the most important and highly conserved regulators of growth and development (Xiong et al., 2013). Three concentrations of RAP (SlTOR inhibitor) (1 μM, 5 μM and 10 μM) were employed to measure the germination rate and seedling height under different treatments. The results showed that the germination rate of tomato seeds and plant height were reduced by RAP treatment (**Figures S4A, B**). The 10 μM RAP concentration was superior to other concentrations, so 10 μM RAP was selected for further testing. Three MHY1485 (SlTOR activator) concentrations (1 μM, 5 μM and 10 μM) were tested. The results indicated that all concentrations could promote seed germination and seedling growth (**Figures S4C, D, E**) and accelerate relative hypocotyl length and root length (**Figures S4F, G**). However, overall, the 5 μM MHY1485 concentration was superior to other concentrations. Therefore, subsequent experiments were carried out using 5 μM MHY1485.

**Figure 4:**
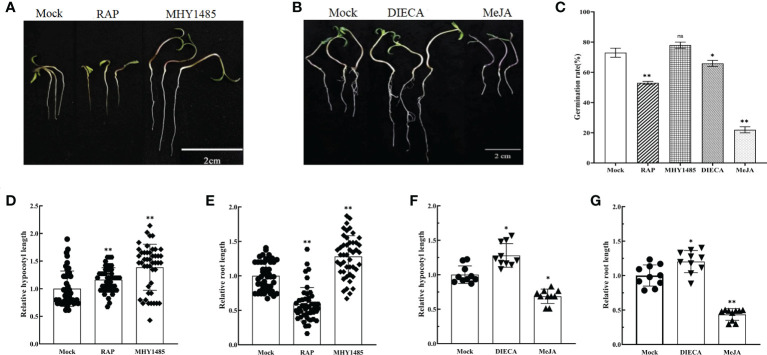
Effects of external application of RAP, MHY1485, DIECA and MeJA on tomato germination, relative hypocotyl length and root length. **(A)** Effects of external application of RAP and MHY1485 on tomato seedlings. **(B)** Effects of external application of DIECA and MeJA on tomato seedlings. **(C)** Effects of external application of RAP, MHY1485, DIECA and MeJA on tomato germination rate. **(D)** Effects of external application of RAP and MHY1485 on tomato relative hypocotyl length. **(E)** Effects of external application of RAP and MHY1485 on tomato relative root length. **(F)** Effects of external application of DIECA and MeJA on tomato relative hypocotyl length. **(G)** Effects of external application of DIECA and MeJA on tomato relative root length. Errors are standard deviations for three biological replicates (n=3). Differences between means were analyzed for significance using Student’s *t* test, *P < 0.05, **P < 0.01. ns, no significant difference.

The treatments with RAP (10 μM) and MHY1485 (5 μM) were used for further experiments. The results demonstrated that RAP inhibited the growth of tomato seedlings, but promoted by MHY1485 (**Figure 4A**). Moreover, the seed germination, relative hypocotyl elongation and root elongation of tomato were significantly inhibited by MeJA treatment but promoted by DIECA (**Figures 4B–G**).

**Figure 5:**
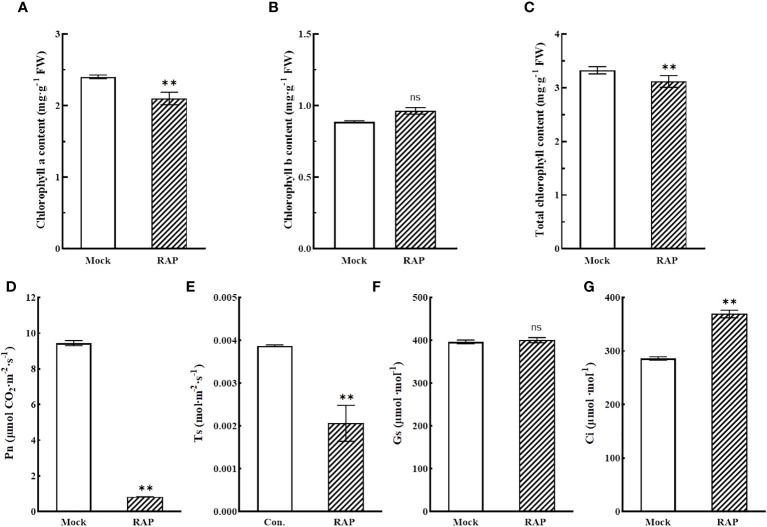
Changes in chlorophyll contents and photosynthetic indexes in leaves of tomato seedlings treated with RAP. **(A)** Chlorophyll a content in tomato leaves after RAP treatment. **(B)** Chlorophyll b content in tomato leaves after RAP treatment. **(C)** Total chlorophyll content in tomato leaves after RAP treatment. **(D)** Net photosynthetic rate in tomato leaves after RAP treatment. **(E)** Transpiration rate in tomato leaves after RAP treatment. **(F)** Stomatal conductance in tomato leaves after RAP treatment. **(G)** Intercellular carbon dioxide concentration in tomato leaves after RAP treatment. Errors are standard deviations for three biological replicates (n=3). Differences between means were analyzed for significance using Student’s *t* test, **P < 0.01. ns, no significant difference.

The photosynthetic changes of tomato seedlings were measured by a photosynthesis-testing instrument. When tomato seedlings were treated with RAP (10 μM), chlorophyll a and total chlorophyll (**Figures 5A, C**), the net photosynthetic rate (Pn) (**Figure 5D**) and the transpiration rate (Ts) (**Figure 5E**) of leaves were significantly reduced, while the changes in chlorophyll b (**Figure 5B**) and stomatal conductance (Gs) (**Figure 5F**) were not significant; however, the intercellular carbon dioxide concentration (Ci) was prominently increased (**Figure 5G**). The results suggested that the photosynthesis of tomato seedlings was inhibited after that RAP inhibited the TOR signaling pathway, but this change was caused by non-stomatal factors.

Under the synergistic treatment of MeJA and RAP, the TOR inhibitor RAP and jasmonic acid MeJA synergistically inhibited the growth of tomato seedlings, and the hypocotyl length and root length were significantly reduced (**Table 1**, **Figure S5**).

**Table 1:** Effect of treatment with MeJA and RAP on tomato seedling growth.

Treatment	Hypocotyl length (cm)	Root length (cm)
Mock	5.558 ± 0.235a	8.057 ± 0.625a
MeJA	3.258 ± 0.228b	3.582 ± 0.637c
RAP	3.510 ± 0.243b	4.330 ± 0.437b
MeJA+RAP	2.327 ± 0.211c	3.118 ± 0.565c

Mock, Control group; MeJA, MeJA treatment; RAP, RAP treatment; MeJA+RAP, Treatment with both MeJA and RAP. Different letters indicate significant differences among treatments. Multiple comparisons of means were performed using Tukey's test at the 0.05 significance level.

**Figure 6:**
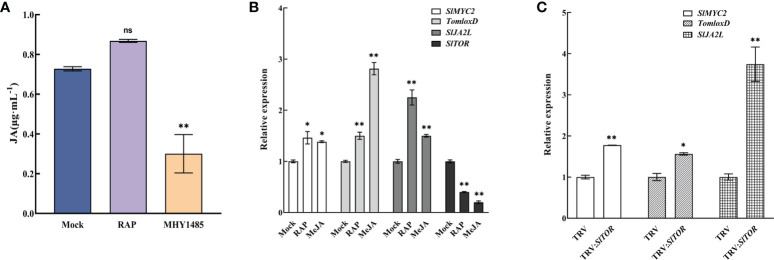
Crosstalk between JA signaling and TOR signaling pathways. **(A)** Endogenous JA content in tomato leaves after treatment with RAP and MHY1485. Mock, Control group. RAP, RAP treatment. MHY1485, MHY1485 treatment. **(B)** Expression of *SlMYC2*, *TomloxD*, *SlJA2L* and *SlTOR* after RAP and MeJA treatment. Mock, Control group. RAP, RAP treatment. MeJA, MeJA treatment. **(C)** Expression of *SlMYC2*, *TomloxD* and *SlJA2L* in TRV : *SlTOR*. TRV, only infiltrated with empty vectors. TRV : *SlTOR*, *SlTOR*-silenced lines. Errors are standard deviations for three biological replicates (n=3). Differences between means were analyzed for significance using Student’s *t* test, *P < 0.05, **P < 0.01. ns, no significant difference.

Further study showed that TOR affected the synthesis of endogenous JA. The content of JA in tomato seedlings was determined after treatment with the TOR inhibitor RAP and activator MHY1485. The results showed that RAP treatment increased the content of endogenous JA, but MHY1485 treatment significantly reduced the amount of JA (**Figure 6A**), indicating that TOR negatively regulated the synthesis of endogenous JA.

Then, RAP and MeJA treatment (**Figure 6B**) and TRV : *SlTOR* lines (**Figure S6**) were used to identify the expression of *SlMYC2* gene and downstream response genes in the JA signaling pathway. The results showed that *SlMYC2*, *TomLoxD* (JA synthesis gene) and *SlJA2L* (*SlMYC2* downstream target gene) were significantly upregulated after TOR signaling was suppressed by RAP and SlTOR silencing (**Figures 6B, C**). They were also upregulated by increasing MeJA, but *SlTOR* expression was downregulated by RAP and MeJA treatment, suggesting an interaction between TOR and JA signaling, which jointly regulated the growth of tomato seedlings.

### SlMYC2 activated *SlTOR* expression by directly binding to the *SlTOR* promoter

**Figure 7:**
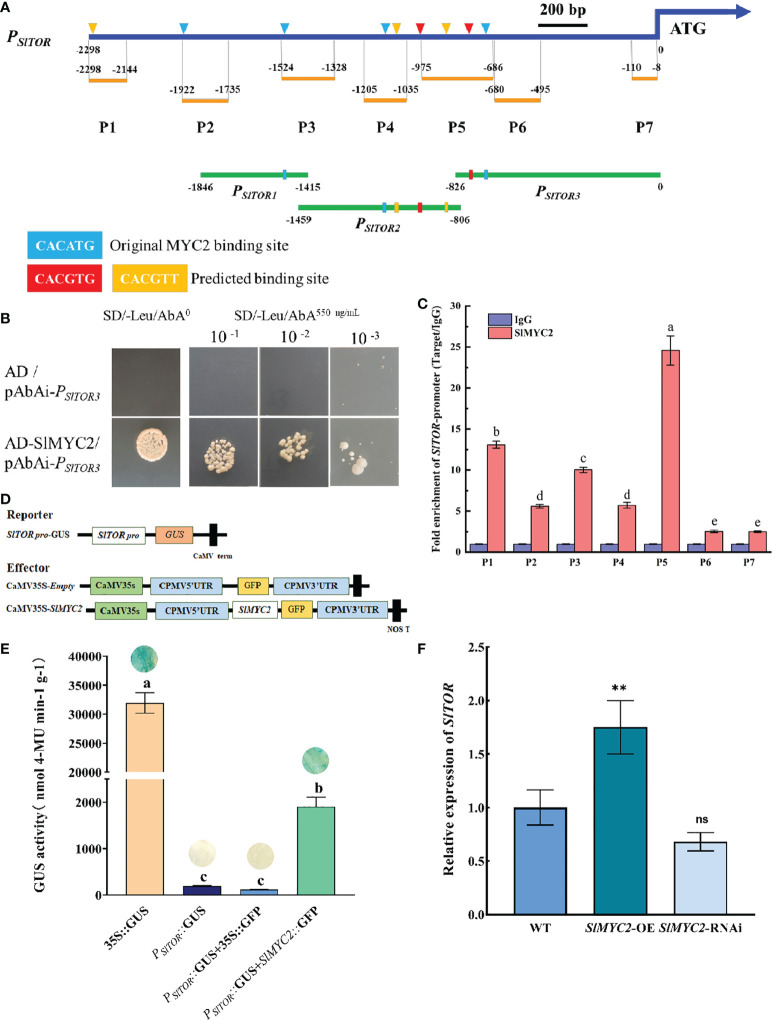
Interaction between SlMYC2 and the *SlTOR* promoter. **(A)** Schematic diagram of segments of *SlTOR* promoter in yeast one-hybrid and ChIP assays. *P_SlTOR1_*, *P_SlTOR2_* and *P_SlTOR3_* are segments of the *SlTOR* promoter in yeast one-hybrid assays, P1-P7 are segments of the *SlTOR* promoter in ChIP assays. CACATG is the original MYC2 binding site, CACGTG and CACGTT are the predicted binding sites which are predicted by PlantCARE. **(B)** Yeast one-hybrid assay. Take 4 μL of bacterial solutions of different concentrations and spread it on a 550 ng·μL^-1^ AbA SD/-Leu plate to observe the yeast growth. **(C)** Schematic representation of the *SlTOR* promoter and chromatin immunoprecipitation (ChIP)–qPCR assays showing the binding of *SlMYC2*-OE to the *SlTOR* promoter upstream of the ATG start codon. P1-P7 represent partitions of the *SlTOR* promoter. Each ChIP value is normalized to its respective input DNA value, and enrichment is shown as the percentage of input. Error bars represent the SEM from three biological replicates. **(D, E)** Analysis of the *SlTOR* promoter activation by SlMYC2 using transient GUS activity assays. A 2-kb *SlTOR* upstream DNA fragment driving GUS was used as the reporter. The effectors were indicated in **(D)**. Measurement of transient GUS activity assay is shown in **(E)**. **(F)** Expression of *SlTOR* after SlMYC2 overexpression and silencing. Errors are standard deviations for three biological replicates (n=3). Differences between means were analyzed for significance using Student’s *t* test, *P < 0.05, **P < 0.01. ns, no significant difference. Different letters indicate significant differences among treatments. Multiple comparisons of means were performed using Tukey's test at the 0.05 significance level.

Further bioinformatic analysis revealed that there were several predicted MYC2 binding elements existed in the *SlTOR* promoter (**Figure 7A** and **Table S2**), suggested that SlMYC2 might bind to the promoter of *SlTOR* and activate its expression *in vivo*. The binding ability of SlMYC2 to *SlTOR* promoter was firstly verified by yeast one-hybrid (Y1H) assay. In Y1H assay, according to the positions of the predicted elements, we divided the *SlTOR* promoter into three fragments: *P_SlTOR1_*, *P_SlTOR2_* and *P_SlTOR3_* (**Figure 7A**). The results showed that yeast cells could growth under 550 ng·μL^-1^ AbA, indicating a potential interaction between SlMYC2 and *P_SlTOR3_*. However, pAbAi-*P_SlTOR1_* and pAbAi-*P_SlTOR2_* were self-activated under the same AbA concentration (**Figure 7B** and **Figure S7**). To further confirm SlMYC2 bound to the *SlTOR* promoter *in vivo*, we performed chromatin immunoprecipitation (ChIP) experiments using *SlMYC2*-OE seedlings. We divided the *SlTOR* promoter into 7 segments for ChIP experiments (**Figure 7A**), and the details of the segments were shown in the **Figures 7A** and **Table S2**. The ChIP results showed that each fragment of the *SlTOR* promoter was enriched in some degree, but highest enrichment occured at P5 segment which located in *P_SlTOR3_* (**Figure 7C**), suggesting that potential binding site of SlMYC2 to *SlTOR* promoter might locate in -686 to -826 of the ATG upstream. Both Y1H and ChIP assays suggested that SlMYC2 bound to *SlTOR* promoter directly. To assay the activation effect of SlMYC2 on *SlTOR* transcription *in vivo*, transient transfections with different reporter and effector vectors were performed respectively. In the GUS histochemical staining assay, transformations with *P_SlTOR_*::GUS and 35S::GFP were used as controls (**Figures 7D, E**). The staining results showed that GUS signal could not been observed when transformed with *P_SlTOR_*::GUS and 35S::GFP; however, it was appeared when co-transformed with both *P_SlTOR_*::GUS and SlMYC2::GFP (**Figure 7E**). GUS enzyme activity analysis also supported the staining result. The GUS activity was significantly higher than those of the controls (**Figure 7E**), suggesting that SlMYC2 regulated the transcription of *SlTOR*. Moreover, we also found that the expression of *SlTOR* was obviously upregulated in the *SlMYC2* overexpression line (**Figure 7F**). Taken together, SlMYC2 might mediate the crosstalk between the JA and TOR signaling pathways through directly binding to the *SlTOR* promoter and activating its expression.

## Discussion

### JA signaling and the transcription factor SlMYC2 contributed to growth and fruit quality in tomato

The growth and development of seedlings are of great importance for obtaining vigorous seedlings and improving yield, quality and resistance. The growth and development of plants are regulated by many plant hormones. Recent studies have shown that JA plays an important role in the regulation of plant growth and development. The phytohormone JA regulates a wide range of biological processes (Chini et al., 2016; Huang et al., 2017; Li et al., 2021a; Song et al., 2022).

Jasmonates (JAs), which consist of jasmonic acid and its derivatives, such as methyl jasmonate (MeJA) and jasmonoyl-isoleucine, play multiple roles in growth and development as well as biotic and abiotic stress responses. Numerous studies have shown that JA is involved in root hair formation, stamen development, flowering, leaf senescence, anthocyanin biosynthesis and photosynthetic carbon fixation (He et al., 2002; Li et al., 2014; Qi et al., 2015; Ding et al., 2018; Wang et al., 2019; Han et al., 2020).

JA is both a hormone and a signaling molecule. MYC2 is a master regulator in the JA signaling pathway that is widely present in plants and animals (Zhang et al., 2015; Breeze, 2019) and has multiple regulatory functions (Chini et al., 2007; Sheard et al., 2010; Li et al., 2021b). MYC2 is an essential helix−loop−helix transcription factor that plays a critical role in the JA signaling pathway and is involved in a variety of plant growth and stress resistance processes. However, the effects of activation and inhibition of JA signaling, and enhancement and silencing of *SlMYC2* on the growth and photosynthesis of tomato seedlings, especially the regulation of tomato fruit quality, have not been systematically investigated.

The formation of fruit quality is inseparable from the growth of tomato seedlings. Only when the plants maintain continuous and healthily growth, can they provide sufficient nutrients for normal fruit development and maturation. To investigate the role of JA and its key transcription factor MYC2 in the growth of tomato seedlings, MeJA and DIECA were used as treatments, and *SlMYC2*-OE and *SlMYC2*-RANi lines were used to measure the growth and developmental indexes of tomato seedlings. The results showed that MeJA treatment and the *SlMYC2* overexpression inhibited the growth, development and photosynthesis of tomato seedlings but increased the root–shoot ratio. Increasing MeJA or the overexpression of *SlMYC2* inhibited the growth of tomato seedlings to some extent, but resulted in vigorous seedlings, indicating that JA signaling played a role in the growth and development of tomato seedlings.

Some studies showed that application of MeJA during preharvest and postharvest stages could enhance fruit antioxidant capacity and phenolic content, thereby extending the shelf life and improving fruit quality (Tzortzakis, 2007; Liu et al., 2018). The insignificant variation in lycopene content in fruits of lines with different jasmonic acid content indicates that the regulation of lycopene accumulation by jasmonic acid may be dependent on specific concentrations (Liu et al., 2012). Some experiments also show that the content of lycopene in the fruit of the JA synthetic mutant *spr2* decreases, suggesting a positive correlation between endogenous JA content and lycopene content in fruits. Exogenous application of MeJA can restore the lycopene content in mutant fruits (Min et al., 2018). Using the *SlMYC2*-silenced tomato fruit obtained by VIGS technology as the material, the results also show that SlMYC2 plays a critical role in MeJA-induced fruit chilling resistance, including the components related to fruit quality (Min et al., 2018). The above evidence suggests that JA and MYC2 play important roles in the regulation of tomato fruit quality. However, systematic studies on the changes of JA and SlMYC2 on tomato fruit quality are lacking.

In this research, we systematically studied the effects of JA and MYC2 on the quality of mature tomato fruits. The results showed that the contents of lycopene and carotenoid increased significantly after MeJA (100 μM) treatment and *SlMYC2* overexpression. Moreover, the contents of soluble sugar, protein, total phenol and flavonoids were also increased. MeJA processing and *SlMYC2* overexpression increased the sugar–acid ratio to improve the quality of fruits, and *SlMYC2* overexpression enhanced fruit firmness. Increasing MeJA or the overexpression of *SlMYC2* improved the quality of tomato fruits, and overexpression of *SlMYC2* increased fruit firmness and prolonged shelf life, indicating that JA signaling played an important role in tomato fruit quality.

### Effects of SlMYC2 mediation on tomato growth and development through crosstalk between the JA and TOR signaling pathways

MYC2 is the key transcription factor in the JA signaling transduction pathway and is a critical component of JA signaling that interacts with other signaling pathways (Dombrecht et al., 2007; Altmann et al., 2020). MYC2 can integrate the interaction between JA signaling and other signals to regulate plant growth and development (Breeze, 2019). Target of rapamycin (TOR) is also an important signaling pathway that regulates plant growth and development, playing a central role in integrating metabolic energy and hormone signals (Dobrenel et al., 2016; Li et al., 2022). Through bioinformatic analysis, the binding elements of MYC2 were identified in the promoter of *SlTOR*. Therefore, it was hypothesized that SlMYC2 might be the node that mediated the crosstalk between the JA and TOR signaling pathways.

Studies have shown that JA signaling and TOR signaling are the core signals that regulate plant growth and development, such as seed germination, rhizome elongation, tricarboxylic acid cycle, starch storage and fruit quality (Xiong et al., 2013; Okello et al., 2015; Song et al., 2017). In addition, there may be an interaction between JA signaling and TOR signaling (Göbel and Feussner, 2009; Mosblech et al., 2009; Salem and Giavalisco, 2019). To clarify the role and relationship between JA and TOR signaling in tomato growth and development, tomato seedlings were treated with MHY1485 (an activator of TOR) and RAP (an inhibitor of TOR). When TOR signaling was inhibited by RAP, it increased the level of JA and inhibited the growth and photosynthesis of tomato seedlings, indicating that TOR signaling positively regulated the growth and development of tomato seedlings. Inhibition of TOR signaling and activation of JA signaling synergistically inhibited the tomato germination and seedling growth; that is, JA signaling and TOR signaling might be involved in crosstalk to regulate the growth and development of tomato seedlings. This result was consistent with previous findings (Song et al., 2017). Moreover, endogenous JA content was significantly reduced after activation of TOR signaling by MHY1485. The endogenous JA content increased after RAP inhibition of TOR, suggesting that TOR signaling inhibited JA regulation during plant growth and development by suppressing JA synthesis, and that SlMYC2 might mediate the crosstalk between JA signaling and TOR signaling pathways.

To show the effect of SlMYC2 on *SlTOR* transcription, the transcription of *SlTOR* in *SlMYC2*-silenced and overexpression lines was examined. The results showed that SlMYC2 promoted the accumulation of *SlTOR* transcription. Therefore, we tentatively determined that SlMYC2 could affect SlTOR signaling by regulating *SlTOR* transcription. To explore the mechanism by which SlMYC2 regulates *SlTOR* transcription, GUS staining, GUS enzyme activity and yeast one-hybrid assays were employed. In this study, yeast one-hybrid and ChIP assays obtained the same results that SlMYC2 could directly interact with *SlTOR*. Further analysis found that the highest enrichment occurred at P5 segment which located in *P_SlTOR3_*, and the *P_SlTOR3_* contained the CACGTG and CACATG motifs in P5 segment, suggesting that potential binding sites of SlMYC2 in *SlTOR* promoter might be located in -686 to -826 of the ATG upstream. It was determined that SlMYC2 could activate the transcriptional accumulation of *SlTOR* by binding to the *SlTOR* promoter. This interaction might be one of the reasons why SlTOR regulated JA signaling by regulating SlMYC2.

The transcription factor MYC2 is a key factor in JA accumulation and signaling activation, and its regulation necessarily induces the response to JA signaling pathway (Heim et al., 2003; Chini et al., 2016; Goossens et al., 2016). SlTOR may regulate the response to the corresponding genes of JA by regulating the level of *SlMYC2*. Therefore, TRV-*SlTOR* lines were constructed to detect the expression of JA synthesis genes and SlMYC2 target genes (*TomLoxD* and *SlJA2L*). The results showed that the expression of *SlMYC2* and its target genes was significantly upregulated. The activation of *SlMYC2* and its target genes were significantly enhanced when SlTOR signaling pathway was inhibited, which uncovered that SlMYC2 mediated the interaction between JA and TOR signaling by acting on the promoter of *SlTOR*.

Studies in *Drosophila* and mammals have shown that TOR can activate PP2A to dephosphorylate MYC, leading to a decrease in MYC stability and protein content. When mTOR is inhibited, MYC2 synthesis is blocked and *MYC2* transcription is enhanced (Lee et al., 2017; Shen et al., 2018). However, more plant evidence is needed. In our unpublished data, the accumulation of SlMYC2 target genes and their protein content were significantly activated by the inhibition of TOR signaling. Therefore, subsequent studies will further reveal whether SlTOR regulates JA signaling through SlMYC2.

In conclusion, *SlMYC2* overexpression could significantly inhibit the growth of tomato seedlings but could improve the quality of tomato fruits. Biochemical experiments showed that inhibition of TOR signaling promoted JA synthesis in tomato and increased the amount of JA in the plant, releasing SlMYC2. SlMYC2 combined with the *SlTOR* promoter, thus activating the *SlTOR* gene, which might feedback on the inhibition of SlMYC2 (**Figure S8**). However, the feedback regulation of SlMYC2 by TOR signaling and the effect on JA signaling still need to be further explored. JA is an anti-stress hormone. The level of JA is low in plants under normal conditions, but under stress, JA is elevated to initiate SlMYC2 and activates *SlTOR* in combination with the *SlTOR* promoter; thus, feedback inhibits JA synthesis and *SlMYC2* expression, leading to a balance of growth and stress resistance. Therefore, the present study can lay the foundation for further revealing the mechanism of JA and TOR signaling interactions in regulating plant growth and development as well as stress resistance balance under stress.

## Data availability statement

The original contributions presented in the study are included in the article/**Supplementary Materials**. Further inquiries can be directed to the corresponding authors.

## Author contributions

YZ and HX contributed to the experimental design, tomato planting and sampling, tobacco planting and experimentation, data processing and result analysis and writing. HW, LY, ZY, and XM contributed to the analysis of the data in the experiment. PH, HF, YY, and NC revised the paper. All authors contributed to the article and approved the submitted version.

## Funding

This research was funded by the National Key Research and Development Program Projects of China (2019YFD1000300) and the Key Project of Science and Technology Research of Liaoning Provincial Education Department (LJKZ0631).

## Acknowledgments

We are particularly grateful to Dr. Chuanyou Li (Institute of Genetics and Developmental Biology Chinese Academy of Sciences) for kindly providing the *SlMYC2* mutants. We would like to thank American Journal Experts (www.aje.com) for English language editing.

## Conflict of interest

The authors declare that the research was conducted in the absence of any commercial or financial relationships that could be construed as a potential conflict of interest.

## Publisher’s note

All claims expressed in this article are solely those of the authors and do not necessarily represent those of their affiliated organizations, or those of the publisher, the editors and the reviewers. Any product that may be evaluated in this article, or claim that may be made by its manufacturer, is not guaranteed or endorsed by the publisher.
