# Supplementary figures and images for: SlMYC2 interacted with the SlTOR promoter and mediated JA signaling to regulate growth and fruit quality in tomato
Source: Front Plant Sci. 2022 Oct 27;13:1013445. doi: 10.3389/fpls.2022.1013445 (PMC9647163; doi:10.3389/fpls.2022.1013445)

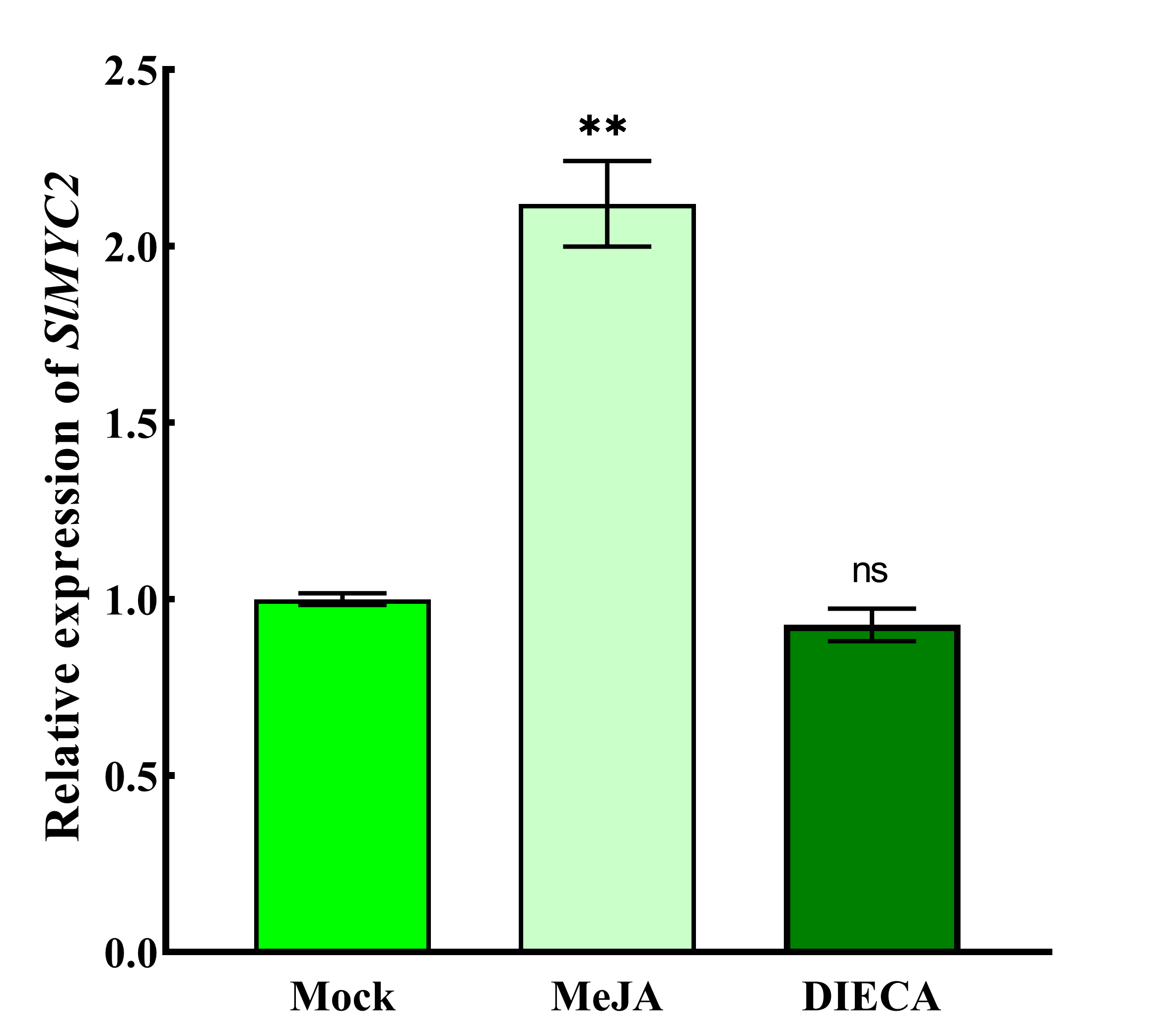

Supplement: Supplementary file 1 [file Image_1.tif]

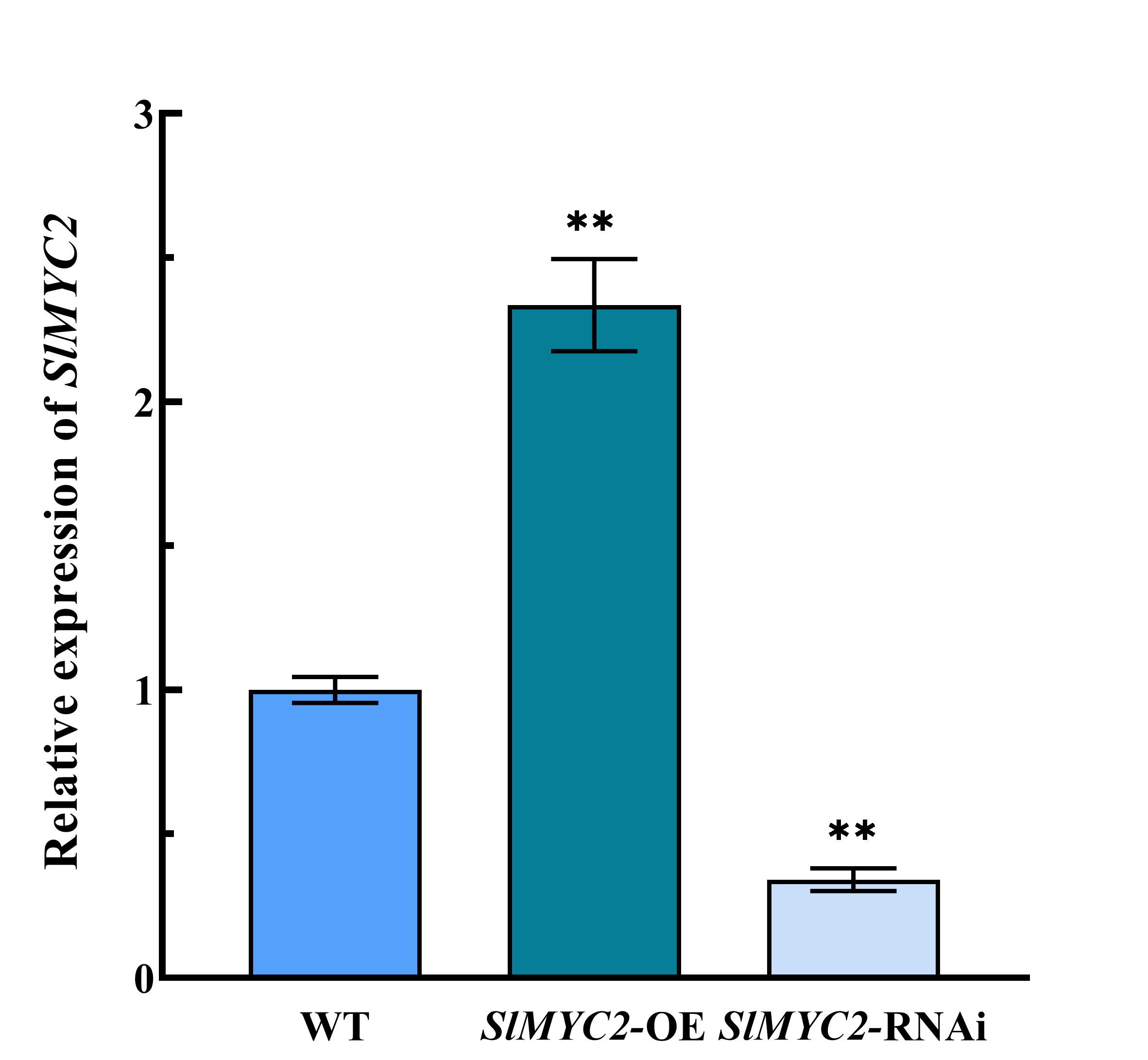

Supplement: Supplementary file 2 [file Image_2.tif]

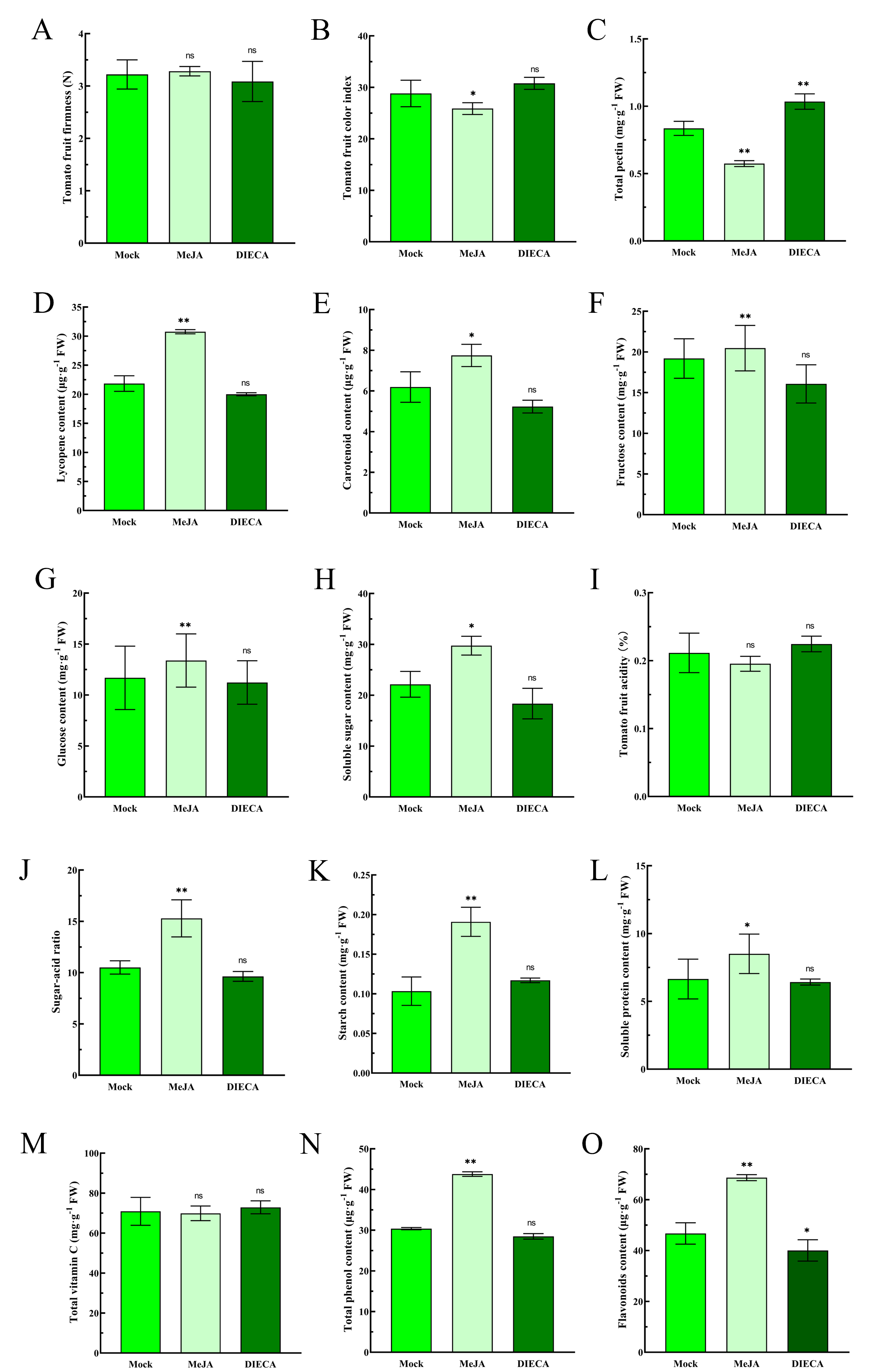

Supplement: Supplementary file 3 [file Image_3.tif]

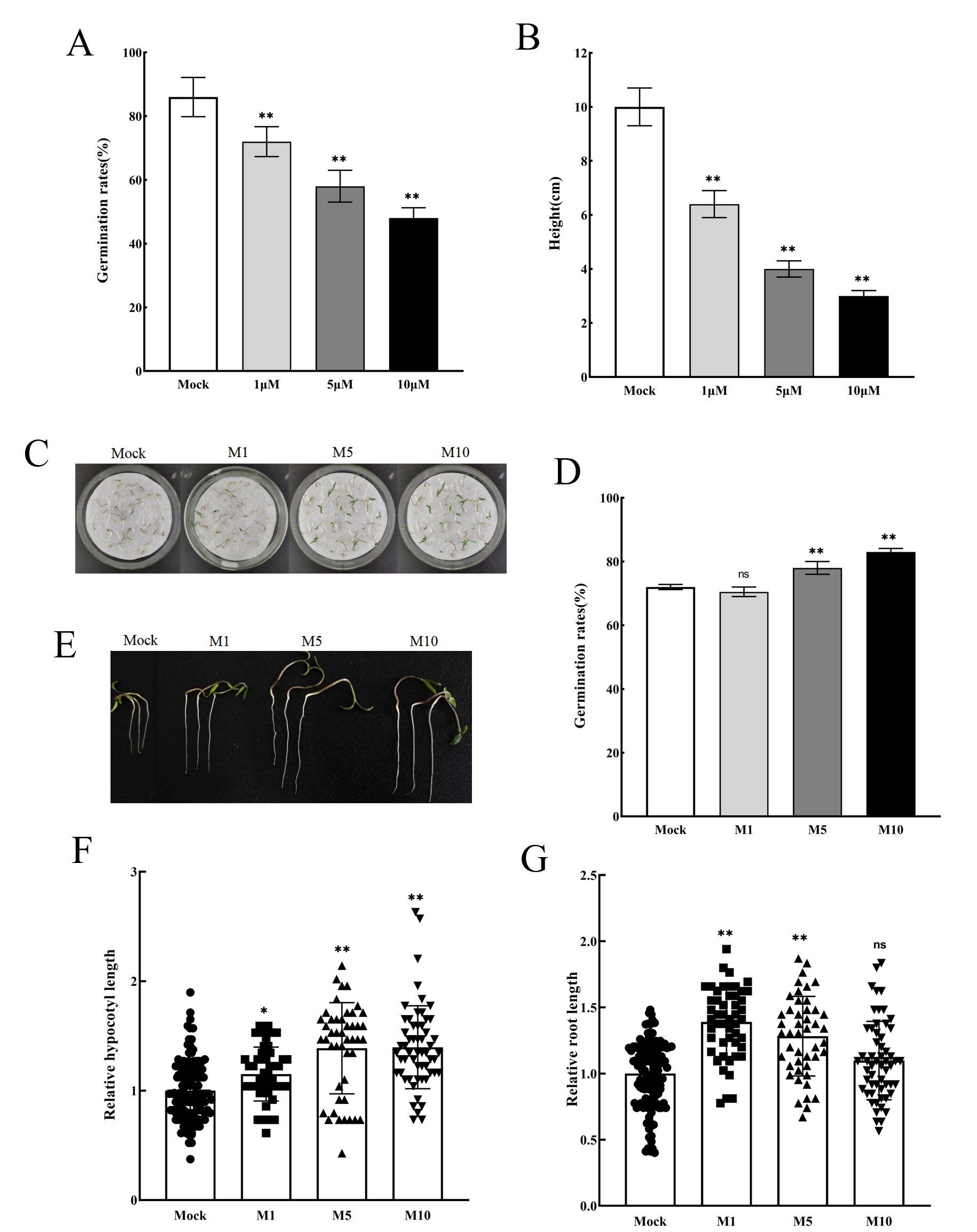

Supplement: Supplementary file 4 [file Image_4.tif]

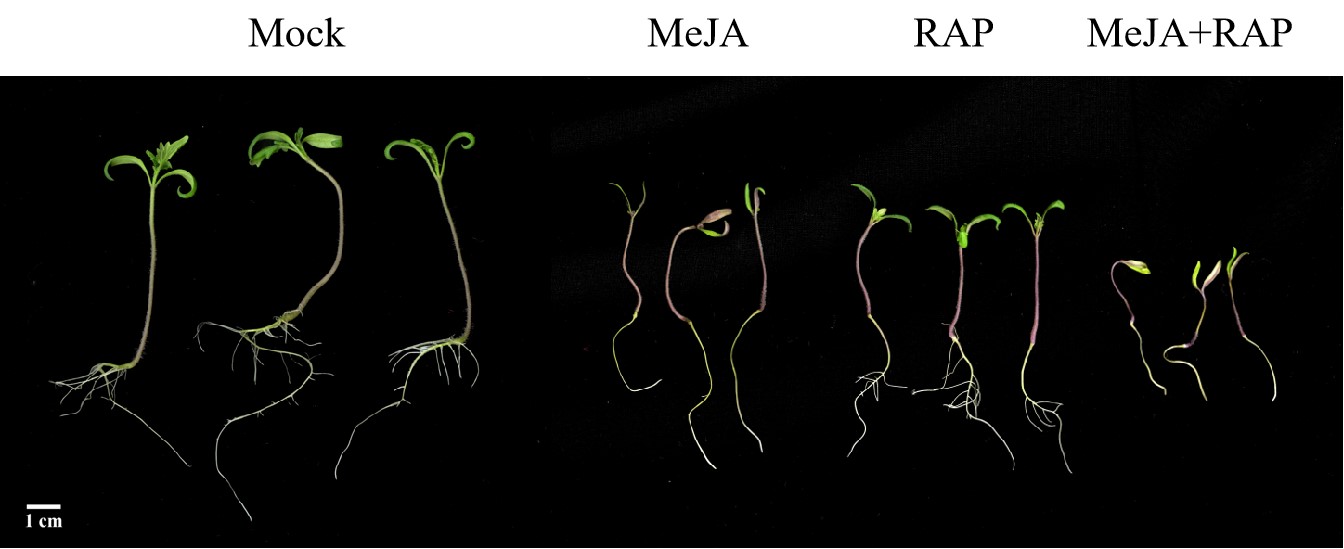

Supplement: Supplementary file 5 [file Image_5.jpeg]

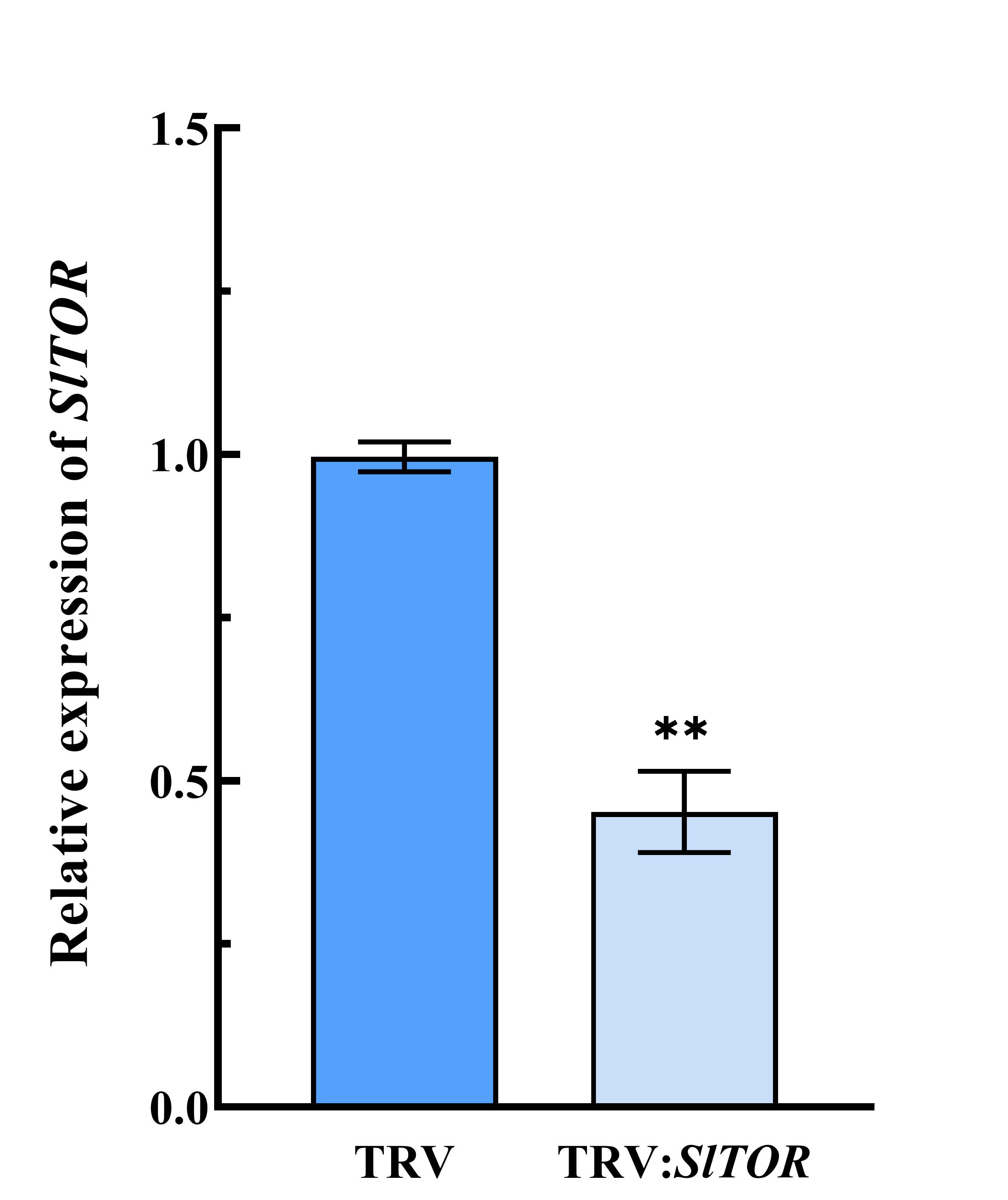

Supplement: Supplementary file 6 [file Image_6.tif]

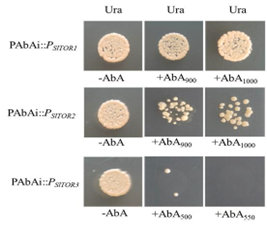

Supplement: Supplementary file 7 [file Image_7.tif]

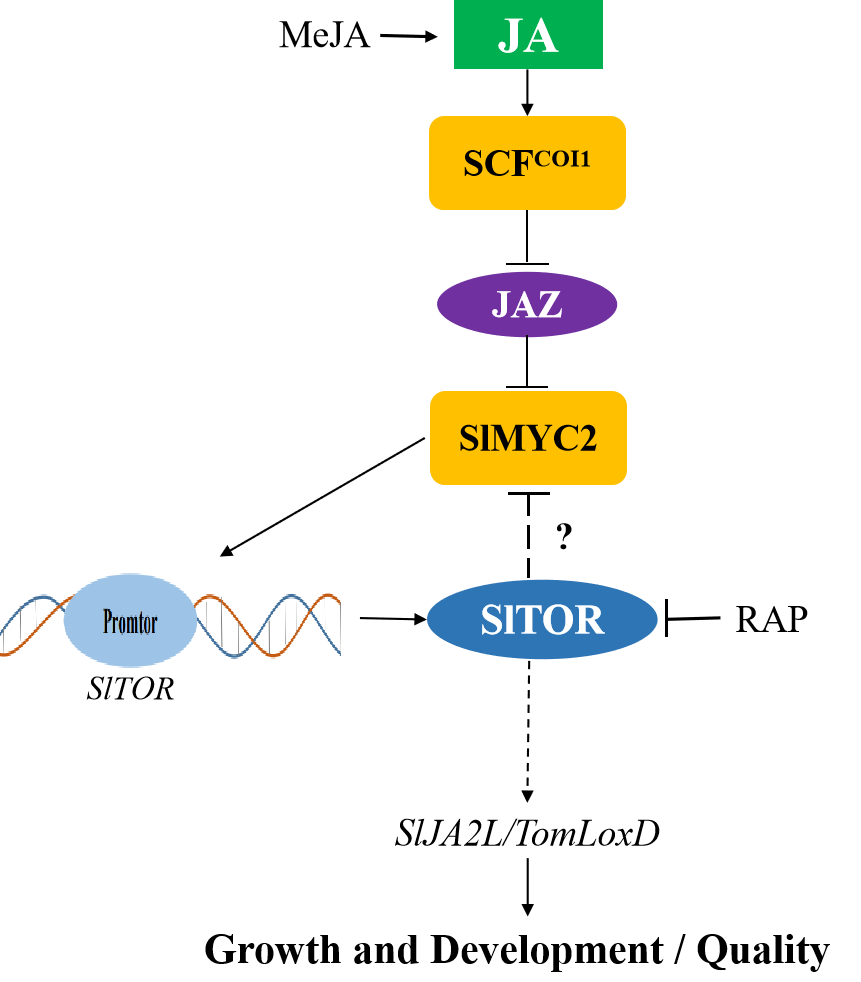

Supplement: Supplementary file 8 [file Image_8.tif]
