# Supplementary material for: SlMYC2 interacted with the SlTOR promoter and mediated JA signaling to regulate growth and fruit quality in tomato
Source: Front Plant Sci. 2022 Oct 27;13:1013445. doi: 10.3389/fpls.2022.1013445 (PMC9647163; doi:10.3389/fpls.2022.1013445)
Supplement: Supplementary file 9 [file DataSheet_1.docx]

FIGURE S1 The expression of *SlMYC2* treated with MeJA and DIECA. Mock, Control group. MeJA, MeJA treatment. DIECA, MeJA treatment. Errors are standard deviations for three biological replicates (n=3). Differences between means were analyzed for significance using Student’s *t* test, **P* < 0.05, ***P* < 0.01. ns, no significant difference.

FIGURE S2 Verification of *SlMYC2* overexpression and silenced lines. *SlMYC2-*OE, *SlMYC2* overexpression. *SlMYC2-*RNAi, *SlMYC2* silenced. Errors are standard deviations for three biological replicates (n=3). Differences between means were analyzed for significance using Student’s *t* test, **P* < 0.05, ***P* < 0.01. ns, no significant difference.

FIGURE S3 Effects of MeJA and DIECA on the quality of tomato fruit. Mock, Control group. MeJA, MeJA treatment. DIECA, DIECA treatment. Errors are standard deviations for three biological replicates (n=3). Differences between means were analyzed for significance using Student’s *t* test, **P* < 0.05, ***P* < 0.01. ns, no significant difference.

FIGURE S4 Effects of the germination rate of tomato seeds and plant height of tomato seedlings treated with RAP and MHY1485. (A) The germination rate of tomato seeds treated with three concentrations of RAP (1 μM, 5 μM and 10 μM). (B) The plant height of tomato seedlings treated with three concentrations of RAP (1 μM, 5 μM and 10 μM). (C-D) The germination rate of tomato seeds treated with three MHY1485 concentrations (1 μM, 5 μM and 10 μM). (E-G) The relative hypocotyl length and root length treated with three MHY1485 concentrations (1 μM, 5 μM and 10 μM). Errors are standard deviations for three biological replicates (n=3). Differences between means were analyzed for significance using Student’s *t* test, **P* < 0.05, ***P* < 0.01. ns, no significant difference.

FIGURE S5 Effects of treatment with MeJA and RAP on tomato seedling growth. Mock, Control group. MeJA, MeJA treatment. RAP, RAP treatment. MeJA+RAP, Treatment with both MeJA and RAP.

FIGURE S6 Verification of TRV:*SlTOR* lines. TRV, only infiltrated with empty vectors. TRV:*SlTOR*, *SlTOR*-silenced lines. Errors are standard deviations for three biological replicates (n=3). Differences between means were analyzed for significance using Student’s *t* test, **P* < 0.05, ***P* < 0.01. ns, no significant difference.

FIGURE S7 Screening of AbA concentration of *SlTOR* promoter for yeast one-hybrid assay.

FIGURE S8 The pattern diagram of crosstalk between JA and TOR signaling pathways mediated by SlMYC2 on the regulation of tomato growth and development. Under normal growth conditions, TOR signaling promoted the growth and development of tomato seedlings. When MeJA was treated, the growth of tomato seedlings was inhibited, but activated SlMYC2. After combining with *SlTOR* promoter and starting the expression of *SlTOR*, feedback inhibits SlMYC2, also reduced the content of JA, and then regulated the growth rate of tomato seedlings to obtain vigorous seedlings.

**TABLE S1** The primer lists used in this study

| **Name** | **Primer Sequences** | **Application** |
| --- | --- | --- |
| *SlMYC2*-F | GGTTGATGAAGAAGTTACCGACAC | qRT-PCR |
| *SlMYC2*-R | CACGCCGTTAGCTGAAGGA |  |
| *SlTOR*-F | CAGGCGATCCGTTATCCAGT |  |
| *SlTOR*-R | AATAGCCCTCAATGCTCCCAG |  |
| *TomLoxD*-F | GTAGTTACAGTAAGGAACAAGAACAAGG |  |
| *TomLoxD*-R | CCCGAAATTCGAGTCCACAA |  |
| *SlJA2L*-F | CACCAATCAGTATCACAATCACAAC |  |
| *SlJA2L*-R | AACTCCTCCTTCAAACTTAACTTCAC |  |
| P*_SlTOR_*-GUS-F | ACGACGGCCAGTGCCAAGCTTCGACAAAATGATTGACAGTAACATG | GUS |
| P*_SlTOR_*-GUS-R | TCAGAATTCGGATCCGGTACCTAACTCTCTACAACATACACGCAGAAC |  |
| *SlMYC2*-GFP-F | CTGTTGATACATATGATGACTGAATACAGCTTGCCCAC |  |
| *SlMYC2*-GFP-R | CATGGATCCGGTACCGTGTGTTTCAGCAATTTTCGA |  |
| pAbAi-*SlTORp1*-F | CTTGAATTCGAGCTCGGTACCGTATGTTTATCTCGTAATACCCCGA | Yeast one-  hybrid |
| pAbAi-*SlTORp1*-R | AGCACATGCCTCGAGGTCGACGGTATTCATACGGGAAAAATCATC |  |
| pAbAi-*SlTORp2*-F | CTTGAATTCGAGCTCGGTACCCGGACTTGCCTAGCTGTTAGG |  |
| pAbAi-*SlTORp2*-R | AGCACATGCCTCGAGGTCGACGTCAGGGAGAATCACACGAGATAA |  |
| pAbAi-*SlTORp3*-F | CTTGAATTCGAGCTCGGTACCCTCGTGTGATTCTCCCTGACAA |  |
| pAbAi-*SlTORp3*-R | AGCACATGCCTCGAGGTCGACTGGCAGCCATGTTTTTAACTCT |  |
| AD-SlMYC2-F | GCCATGGAGGCCAGTGAATTCATGACTGAATACAGCTTGCCCAC |  |
| AD-SlMYC2-R | CAGCTCGAGCTCGATGGATCCTTAGTGTGTTTCAGCAATTTTCGA |  |
| *P1-F* | CGATACATCATTATCACGTTATTCG | ChIP-qPCR |
| *P1-R* | GCACCAAAATGATCAATTGTCAG |  |
| *P2-F* | CACTCACATGATTTGTGATATCGTG |  |
| *P2-R* | CGCACGAAAATGATCAATTATCA |  |
| *P3-F* | CGGACTTGCCTAGCTGTTAGG |  |
| *P3-R* | CGCACGAAAATGATCAATTGTC |  |
| *P4-F* | CATCCGCATTAATCAGTAACGTC |  |
| *P4-R* | CAGCTAGGCAATTACAAATAACGTG |  |
| *P5-F* | CGTTCATTTTCCACGTGACTTATA |  |
| *P5-R* | GGGAGAATCACACGAGATAAAATT |  |
| *P6-F* | TGACAAAATTTATTCATCCGCATT |  |
| *P6-R* | CTCTCTACAACATACACGCAGAACA |  |
| *P7-F* | ACAAATGCGTGTCTACAATTTCTCT |  |
| *P7-R* | GTGGTTGCAACTGGATAACGG |  |

Note: Accession numbers (Sol Genomics Network data libraries). *SlMYC2* (Solyc08g076930), *SlTOR* (Solyc01g106770), *TomLoxD* (Solyc03g122340), *SlJA2L* (Solyc07g063410).

**TABLE S2** Bioinformatics analysis revealed the presence of binding elements of MYC2 in the *SlTOR* promoter

| **Cis-element** | **Origin of plant** | **Locus** | **Sequence (5‘-3’)** |
| --- | --- | --- | --- |
| G-box | *Solanum lycopersicum* | -693, -1098, -1506, -1912 | CACATG |
| G-box | *Arabidopsis thaliana* | -854,-1054,-2278 | CACGTT |
| G-box | *Arabidopsis thaliana* | -762,-958 | CACGTG |

Note: CACATG is the MYC2 binding motif on the *SlTOR* promoter, and the number represents the position of the preceding motif of the ATG upstream.
